# Preoperative Systemic Immune-Inflammation Index (SII) for Predicting the Survival of Patients with Stage I-III Gastric Cancer with a Signet-Ring Cell (SRC) Component

**DOI:** 10.1155/2020/5038217

**Published:** 2020-05-22

**Authors:** Ziyu Zhu, Xiliang Cong, Rui Li, Xin Yin, Chunfeng Li, Yingwei Xue

**Affiliations:** ^1^Department of Gastrointestinal Surgery, Harbin Medical University Cancer Hospital, Harbin, China; ^2^Department of Digestive Disease Center, Qiqihar First Hospital, Qiqihar, China

## Abstract

**Background:**

Recently, a novel systemic immune-inflammation index (SII) based on peripheral lymphocytes, neutrophils, and platelets has been reported to be correlated with patient prognosis in several malignancies, including gastric cancer. However, the prognostic value of the SII for gastric cancer patients with a signet-ring cell (SRC) component has not yet been reported. In this study, we aimed to assess the prognostic value of the SII in gastric cancer patients with an SRC component after curative resection.

**Methods:**

This study was a retrospective analysis of 512 GC patients with an SRC component who underwent curative resection. The prognostic value of the SII was analyzed by the Kaplan-Meier method and Cox proportional hazards regression model.

**Results:**

In our study cohort, an optimal cut-off value for the SII of 527 was used to stratify patients with gastric cancer (GC) into low (<527) and high SII (≥527) groups. Our study indicated that a high SII (≥527) was significantly correlated with a large tumor size (*p* < 0.001), infiltration of serosa (*p* < 0.001), lymph node metastasis (*p* < 0.001), and advanced TNM stage (*p* < 0.001). Univariate and multivariate analyses further demonstrated that a low SII was correlated with better clinical outcome and was an independent prognostic predictor in GC patients with an SRC component. Furthermore, the SII retained prognostic value in the subgroup analysis, including subgroup of different TNM stages and pure or mixed signet-ring cell carcinomas (SRCCs).

**Conclusion:**

The SII is a simple, promising, and practical prognostic biomarker for patients with surgically resected mixed SRCC and pure SRCC. The SII could complement current prognostic tools for better treatment planning and stratification of patients.

## 1. Introduction

Gastric cancer (GC) is the third leading cause of death from malignancy and the fifth most common carcinoma worldwide [1]. In the past two decades, obvious progress has been made in diagnosing and treating this lethal carcinoma via biological targeted therapy, chemotherapy, and radiotherapy. The 5-year survival rate for patients with GC remains approximately 30% [2, 3]. Curative resection (R0 resection) remains the best option for patients with GC [4].

In recent years, although the incidence of GC has been reported to be decreasing, the proportion of diffuse-type GC in gastric cancer has been increasing [5, 6]. Correspondingly, the subtypes of the diffuse type of GC have also seen increasing incidence, including pure signet-ring cell cancer (pSRCC) and mixed signet-ring cell cancer (mSRCC) [2, 7].

Signet-ring cell (SRC) gastric cancer is a special type of histopathology in gastric cancer that features intracytoplasmic mucin within tumor cells that pushes the nucleus to the periphery [8]. The classifications developed by Sugano, Ming, and Lauren designate SRCC as “undifferentiated type,” “infiltrative type,” and “diffuse type,” respectively [9–11]. Some studies have demonstrated that SRCC has unique biological behavior and poor prognosis compared to other subtypes [12]. Therefore, research on gastric cancer with an SRC component (pSRCC and mSRCC) has an important value.

Until now, the TNM stage has been a major determinant for treatment planning and prognosis evaluation [13]. However, an accurate TNM stage can be obtained after surgery, and the role of the TNM stage in predicting prognosis is not perfect. Although gene analyses and molecular profiling have shown tremendous potential in instructing patient curative strategies, these technologies are expensive and complex at present [14, 15]. Therefore, the convenient and simple biomarkers in clinical practice that have aided in guiding patient stratification, determining curative strategies, and predicting prognosis have an important value.

In recent years, an increasing number of studies have indicated that inflammation is related to cancer [16]. Noticeably, an increasing number of scholars are paying more attention to inflammatory indexes that can reflect the whole-body state, such as the systemic immune-inflammation index (SII), lymphocyte to neutrophil ratio (NLR), and prognostic nutritional index (PNI) [17, 18]. Recent studies have shown that the SII based on platelets, lymphocytes, and neutrophils as a combination biomarker can be utilized to predict prognosis in patients with pancreatic cancer, breast cancer, and hepatocellular carcinoma [19–21]. This novel comprehensive prognostic parameter combining peripheral platelets, lymphocytes, and neutrophils is superior to individual cell type-based factors in prognostic prediction, perhaps due to its more comprehensive reflection of the balance of immune status and host inflammation. However, the prognostic value of the SII in gastric cancer with an SRC component remains unexplored thus far.

In our study, the primary purpose was to evaluate the prognostic value of the SII in gastric cancer patients with an SRC component who received curative resection (R0 resection).

## 2. Materials and Methods

### 2.1. Patients

Between May 2001 and December 2013, a total of 512 patients who were diagnosed with pSRCC or mSRCC and underwent gastrectomy at Harbin Medical University Cancer Hospital, Harbin, China, were retrospectively analyzed. The inclusion criteria were as follows: (1) patients were diagnosed with pSRCC or mSRCC through pathological examination after radical surgery for gastric cancer (R0 resection); (2) patients received radical surgery for gastric cancer (R0 resection); (3) patients did not receive preoperative neoadjuvant chemotherapy or radiotherapy; (4) patients were not allowed to receive nutrition replacement therapy or any drugs that may affect serum makers before collection of blood samples; and (5) patients had complete clinicopathological and follow-up data. In our study, patients who had immune or hematological disease, died of non-tumor-related causes, or died within one month after the operation were excluded. All blood biochemistry samples were collected within one week before surgery and examined by the laboratory department of Harbin Medical University Cancer Hospital. The results of biochemistry tests and routine blood tests included tests of leukocytes (10^9^/L), neutrophils (10^9^/L), platelets (10^9^/L), lymphocytes (10^9^/L), serum fibrinogen (g/L), serum hemoglobin (g/L), serum prealbumin (g/L), serum albumin (g/L), and serum globulin (g/L). Other clinicopathological factors included sex, age, receipt of total gastrectomy, depth of tumor infiltration, lymph node metastasis, TNM stage [22], pathologic differentiation type [8], tumor size, and tumor location.

In the present study, the SII and NLR were calculated as follows: SII = N × P/L and NLR = N/L, where L, N, and P represent lymphocytes, neutrophils, and platelets, respectively; prognostic nutritional index (PNI) = serum albumin (g/L) + lymphocyte count × 5 (10^9^/L).

### 2.2. pSRCC and mSRCC

In our study, signet-ring cell gastric carcinomas were classified based on the WHO diagnostic criteria. Cases with a relatively large amount of intracytoplasmic mucin (>50% of the tumor volume) within tumors were defined as pSRCCs; cases with a relatively small amount of intracytoplasmic mucin (10%-50% of the tumor volume) within tumors were defined as mSRCCs [7].

### 2.3. Follow-Up

The follow-up of patients was completed with the Gastric Tumor Information Management System V1.0 and the hospital follow-up group (follow-up was every 3 months in the first two years and every 6 months thereafter). The overall survival (OS) time was from the date of operation to the date of death or the date of last follow-up. Follow-up occurred from August 2001 to December 2018.

### 2.4. Statistical Analysis

SPSS 21.0 software (SPSS, Chicago, IL) was used for all statistical analyses. The optimal cut-off levels for prognostic factors were determined by ROC curve analysis. The correlation between the SII and characteristics was tested by a chi-square test. Survival differences were compared by the Kaplan-Meier method and a log-rank test. Multivariate prognosis analysis was performed using a Cox regression model with time-dependent covariates. *p* < 0.05 was considered statistically significant.

## 3. Result

### 3.1. Patient Characteristics

There were 512 patients in our study, with a median age of 55 years. The study included 332 males and 180 females and 68 cases of pSRCC and 444 cases of mSRCC. A total of 133 patients underwent total gastrectomy, and 399 patients underwent partial gastrectomy. A total of 292 patients were diagnosed with stage III disease, and 220 patients were diagnosed with stage I or II disease. There were 340 patients in the low SII (<527) group and 172 patients in the high SII (≥527) group (Table 1). A total of 244 (47.7%) patients received fluorouracil-based postoperative adjuvant chemotherapy. Five years after surgery, 242 patients died.

### 3.2. The Optimal Cut-off Values for Prognostic Factors

**Figure 1:**
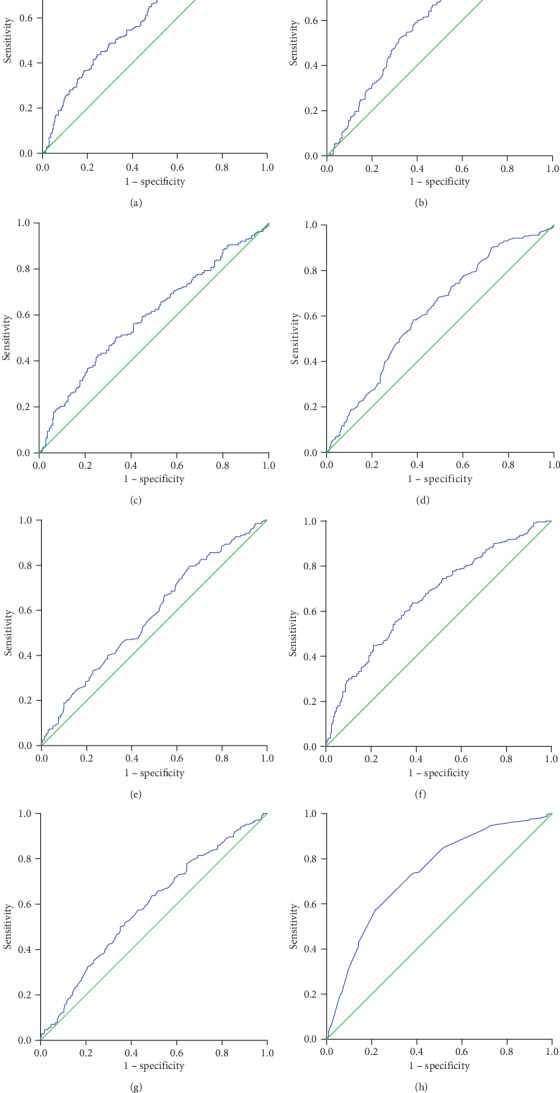
Receiver operating characteristic (ROC) curve analysis of the (a) SII, (b) PNI, (c) NLR, (d) fibrinogen level, (e) hemoglobin level, (f) prealbumin level, (g) albumin level, and (h) tumor size.

Receiver operating characteristic (ROC) curves, using 5-year OS rates as the end-point, for the SII, the PNI, the NLR, leukocyte count, neutrophil count, fibrinogen level, hemoglobin level, prealbumin level, albumin level, globulin level, and tumor size were generated. The area under curve (AUC) values for the SII, the PNI, the NLR, leukocyte count, neutrophil count, fibrinogen level, hemoglobin level, prealbumin level, albumin level, globulin level, and tumor size were 0.615 (*p* < 0.001), 0.615 (*p* < 0.001), 0.593 (*p* < 0.001), 0.502 (*p* = 0.941), 0.539 (*p* = 0.130), 0.617 (*p* = <0.001), 0.578 (*p* = 0.002), 0.662 (*p* < 0.001), 0.587 (*p* = 0.001), 0.505 (*p* = 0.855), and 0.735 (*p* < 0.001), respectively (Figure 1). The optimal cut-off level based on 5-year OS was determined to be 527 for the SII, 48.73 for the PNI, 2.2 for the NLR, 6.17 for leukocyte count, 3.27 for neutrophil count, 3.06 for fibrinogen level, 121.2 for hemoglobin level, 234.5 for prealbumin level, 42.5 for albumin level, 29.9 for globulin level, and 4.75 for tumor size (Table 2).

### 3.3. Correlation between the SII and Patient Characteristics

There were 340 patients in the low SII (<527) group and 172 patients in the high SII (≥527) group. There was a significant difference between the low SII (<527) group and the high SII (≥527) group in terms of tumor size (*p* < 0.001), infiltration of serosa (*p* < 0.001), lymph node metastasis (*p* < 0.001), TNM stage (*p* < 0.001), leukocyte count (*p* < 0.001), neutrophil count (*p* < 0.001), serum hemoglobin level (*p* < 0.001), plasm fibrinogen level (*p* < 0.001), serum prealbumin level (*p* = 0.005), the PNI (*p* < 0.001), and the NLR (*p* < 0.001). We found that the patients in the high SII group seemed to have a larger tumor size, higher TNM stage, higher leukocyte count, higher neutrophil count, higher plasma fibrinogen level, and higher NLR than those in the low SII group. The high SII group seemed to have patients with higher serum hemoglobin levels, higher serum prealbumin levels, and higher PNI values than the low SII group (Table 3).

### 3.4. Univariate and Multivariate Survival Analyses

In the univariate analysis, we concluded that patients with younger age (*p* = 0.007), smaller tumor diameter (*p* < 0.001), shallower depth of tumor invasion (*p* < 0.001), no lymph node metastasis (*p* < 0.001), less advanced TNM stage (*p* < 0.001), a primary site within the lower third of the stomach (*p* < 0.001), low neutrophil level (*p* = 0.046), high hemoglobin level (*p* < 0.001), low plasma fibrinogen level (*p* < 0.001), high prealbumin level, high serum albumin level (*p* < 0.001), low NLR (*p* = 0.006), high PNI (*p* < 0.001), low SII (*p* < 0.001), and no receipt of total gastrectomy (*p* < 0.001) had better prognosis (Table 4). In subsequent multivariate analysis, the results demonstrated that SII (HR (95% CI): 1.634 (1.121-2.382), *p* = 0.011), depth of tumor invasion (HR (95% CI): 1.995 (1.562-2.548), *p* < 0.001), lymph node metastasis (HR (95% CI): 1.481 (1.282-1.711), *p* < 0.001), and receipt of total gastrectomy (HR (95% CI): 0.447 (0.324-0.618), *p* < 0.001) were independent risk factors for gastric cancer patients with an SRC component (Table 5).

### 3.5. The SII and OS in Subgroup Analysis

**Figure 2:**
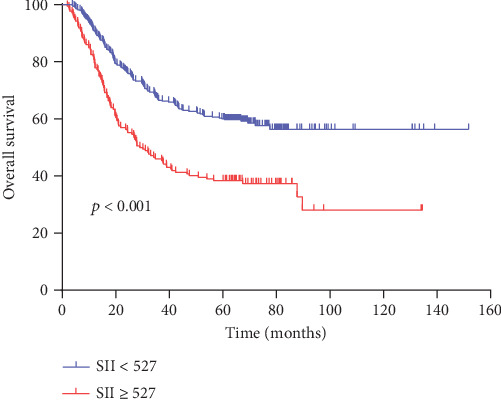
Kaplan-Meier analysis of overall survival (OS) for the SII in all patients with gastric cancer.

**Figure 3:**
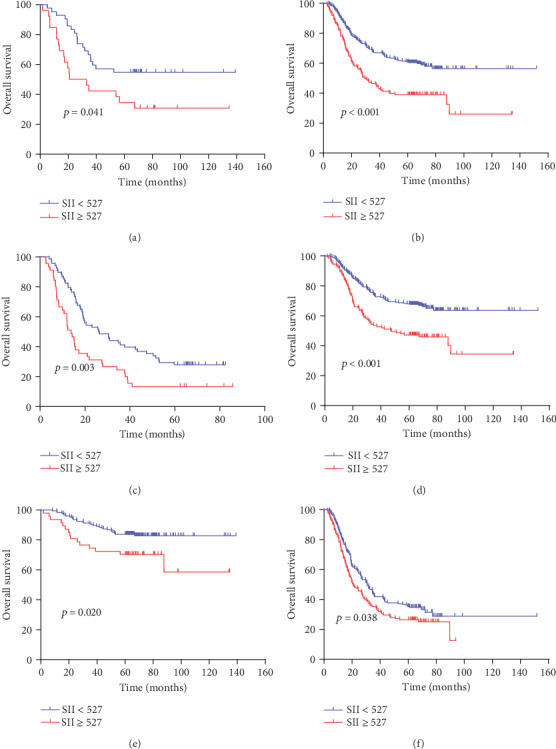
Kaplan-Meier analysis of OS for the SII in patients with gastric cancer. (a) Kaplan-Meier analysis of OS for the SII in patients with gastric cancer in the pSRCC group; (b) Kaplan-Meier analysis of OS for the SII in patients with gastric cancer in the mSRCC group; (c) Kaplan-Meier analysis of OS for the SII in patients with gastric cancer in the total gastrectomy resection group; (d) Kaplan-Meier analysis of OS for the SII in patients with gastric cancer in the nontotal gastrectomy resection group; (e) Kaplan-Meier analysis of OS for the SII in patients with gastric cancer in stages I+II; (f) Kaplan-Meier analysis of OS for the SII in patients with gastric cancer in stage III.

The 5-year OS rates between the low SII group and the high SII group were significantly different (60.0% vs. 38.4%, respectively, *p* < 0.001, Figure 2). We further investigated the prognostic value of the SII in pure and mixed SRCC, patients with and without receipt of total gastrectomy, and patients with different TNM stages. A strong relationship between the SII and OS was found in both pure and mixed SRCC (*p* = 0.041 for pSRCC, *p* < 0.001 for mSRCC, Figures 3(a) and 3(b)), in both patients who did and patients who did not receive total gastrectomy (*p* = 0.003 for receipt of total gastrectomy, *p* < 0.001 for no receipt of total gastrectomy, Figures 3(c) and 3(d)), and patients with different TNM stages (*p* = 0.020 for stage I+II, *p* = 0.038 for stage III, Figures 3(e) and 3(f)).

## 4. Discussion

In our study, we determined the prognostic value of the SII in patients with an SRC component who received radical surgery. The results showed that the SII was a prognostic indicator in gastric cancer patients with an SRC component.

Currently, evaluating prognosis in gastric cancer patients is mainly based on TNM staging, which considers characteristics such as histological type, nodal involvement, depth of invasion, distant metastasis, and tumor size, among others [23]. Nevertheless, gastric cancer patients with equivalent clinical and pathological staging may experience different outcomes. The results suggested that other pathological characteristics are related to cancer progression.

In recent years, a few biomarkers were found to be related to poor outcomes in patients with cancer, and they are therefore used to monitor recurrence and to predict prognosis. Increasing studies have shown that the tumor-associated inflammatory response plays a principal role in the progression and development of diverse organ cancers. Therefore, an increasing number of scholars are paying more attention to systemic inflammatory indicators [24, 25]. The SII, which is based on lymphocytes, platelets, and neutrophils, appears to be a powerful prognostic index in a variety of malignancies, including gastric cancer [26–28].

In recent years, a meta-analysis including 7,657 patients from 22 articles showed that a high SII was related to poor survival in patients with a variety of cancers regardless of cut-off value, ethnicity, and sample size [29]. Currently, a meta-analysis of 24 studies (involving a total of 9626 patients) found that a high SII was greatly associated with poor clinical outcomes in patients with gastrointestinal cancers [30]. A study from China showed that preoperative SII was not only an independent prognostic factor for survival in patients with gastric cancer but also significantly associated with OS in different stages [31]. These results strongly support the prognostic value of the SII in gastric cancer.

The mechanism by which the SII affects the occurrence and progression of gastric cancer may include the following aspects. First, neutrophils can promote distant metastasis by triggering both parenchymal and endothelial cells to intensify circulating tumor cell adhesion [32]. In addition, neutrophils can secrete both molecules that lead to DNA damage and substances that promote angiogenesis, such as vascular endothelial growth factor [33]. Second, platelets can promote distant metastasis by inducing epithelial-mesenchymal transition; on the other hand, platelets can serve as a protective “cloak” to defend circulating tumor cells from immune destruction [34]. In cancer cells, the NF-*κ*B and TGF*β*/Smad pathways are activated by direct platelet-tumor cell interactions and platelet-derived TGF*β* effectors, which induce mesenchymal-like transition and cooperate to promote metastasis. Therefore, platelets play a main role in cancer cell metastasis and survival [35]. Third, lymphocytes can induce cytotoxic cell death and cytokine secretion, as well as suppress tumor cell migration and proliferation, to control tumor growth [36]. In addition, a low lymphocyte level was associated with poor survival in cancer, possibly because the host's anticancer immunity is weakened as lymphocyte levels decrease [37].

We found that a higher SII may represent higher neutrophil levels, higher platelet levels, and lower lymphocyte levels. According to the above existing mechanisms, the results suggested that a higher SII fundamentally means weaker immune defense and a stronger inflammatory response in patients with cancer, which leads to poor survival.

In our study, we found that a high SII was related to advanced tumor invasion, lymph node metastasis, advanced TNM stage, and large tumor size in gastric cancer patients with an SRC component. In one study of squamous cell carcinoma of the esophagus, Feng et al. showed that a high SII is related to advanced TNM stage and large tumor size [38]. Similarly, the study results of Huang et al. were also consistent with the results of our study [39]. These results strongly support the close relationship between inflammation and cancer and demonstrate that the inflammatory response parallels tumor progression to a certain degree. By analyzing patients with an SRC component, we found that the 5-year survival rate in the low SII group was significantly higher than that in the high SII group (60.0% vs. 38.4%, *p* < 0.001), and more significantly, the SII was still statistically significant in the multivariate analysis of SRCC patient overall survival. Therefore, the SII is an independent risk factor affecting the prognosis of GC patients with an SRC component. Nie et al. suggested that the SII is an independent prognostic factor for epithelial ovarian cancer [40]. Tao et al. found that compared with a low SII, a high SII was significantly correlated with a lower overall survival rate 5 years after surgery [41]. These results strongly support the conclusions of our study.

Clinical and pathological staging obtained by surgical and postoperative histological examination is still the main index to evaluate the prognosis and survival of patients. However, compared to such staging, the SII is simpler and more convenient to calculate. In addition, the SII is repeatable and generalizable. Therefore, the SII is very applicable in clinical practice.

Significantly, we found in the subgroup analysis that the SII was significantly associated with OS regardless of the type of SRCC (pSRCC or mSRCC), receipt of total gastrectomy (total gastrectomy or no total gastrectomy), and TNM stage (I+II stage or III stage). These results reinforce the value of the SII in the prognostic assessment of GC patients with an SRC component and suggest that it is a complementary method to clinical and pathological TNM staging.

The SII is a comprehensive indicator composed of three elements (neutrophils, platelets, and lymphocytes), which can comprehensively reflect the changes in human physiology, so it has an important clinical value for early detection, determination of treatment, and evaluation of prognosis in GC patients with an SRC component. Although there have been previous studies on the SII and GC, they included patients with all types of gastric cancer, while our study included only GC patients with an SRC component, which enabled a more detailed and accurate identification of more representative prognostic factors for patients with this specific subset of GC. Therefore, our study on whether the SII can be used as a clinical feature to evaluate the prognosis of GC patients with an SRC component is of great significance and value.

Currently, most studies have looked at the SII in patients from different countries and regions. In addition, the SII has been evaluated by different methods, such as analyses of ROC curves, medians, and averages, in previous studies. Therefore, we cannot determine the ideal SII cut-off value. In addition, the mechanisms by which neutrophils, lymphocytes, and platelets affect cancer are also controversial. Whether a preoperative increase in SII suggests promotion or inhibition of peripheral blood neutrophils, lymphocytes, and platelets in the human body is not known. This is also a limitation of this study. Although we searched for the mechanism behind a preoperative SII increase through cell experiments, immunohistochemistry, and animal experiments, we were unable to find it, and prospective, multicenter, and large-sample studies are needed to define and clarify the optimal cut-off value for the SII.

In summary, we believe that gastric cancer patients should be more carefully stratified and that SRCC should be regarded as a disease with its own unique clinical and pathological characteristics. In addition, the SII should be regarded as an important reference and evaluation tool for both early cancer screening in unaffected populations and prognostication of patients with cancer.

In conclusion, we found that a high SII was associated with poor OS in gastric cancer patients with an SRC component. This biomarker may help clinicians to predict patient prognosis.

## Figures and Tables

**Table 1:** Characteristics of 512 patients with gastric cancer with an SRC component.

Variables	Value (%)
Sex	
Men	332 (64.8)
Women	180 (35.2)
Age (years)	
<55	246 (48.0)
≥55	266 (52.0)
SRCC	
pSRCC	68 (13.3)
mSRCC	444 (86.7)
Chemotherapy	
Yes	244 (47.7)
No	268 (52.3)
Tumor size(cm)	
<4.75	228 (44.5)
≥4.75	284 (55.5)
pT	
T_1_	84 (16.4)
T_2_	64 (12.5)
T_3_	62 (12.1)
T_4_	302 (59.0)
pN	
N_0_	166 (32.4)
N_1_	86 (16.8)
N_2_	100 (19.5)
N_3_a	102 (19.9)
N_3_b	58 (11.4)
pTNM	
I+II	220 (43.0)
III	292 (57.0)
Tumor location	
Lower stomach	328 (64.1)
Middle stomach	75 (14.6)
Upper stomach	35 (6.8)
LM stomach	53 (10.3)
MU stomach	7 (1.4)
LMU stomach	14 (2.8)
Leukocyte	
<6.17	292 (57.0)
≥6.17	220 (43.0)
Neutrophil	
<3.27	260 (50.8)
≥3.27	252 (49.2)
Hemoglobin	
<121.2	140 (27.3)
≥121.2	372 (72.7)
Fibrinogen	
<3.06	268 (52.3)
≥3.06	244 (47.7)
Prealbumin	
<234.5	247 (48.2)
≥234.5	265 (51.8)
Albumin	
<42.5	292 (57.0)
≥42.5	220 (43.0)
Globulin	
<29.9	425 (83.0)
≥29.9	87 (17.0)
SII	
<527	340 (66.4)
≥527	172 (33.6)
PNI	
<48.73	176 (34.4)
≥48.73	336 (65.6)
Total gastrectomy	
Yes	113 (22.1)
No	399 (77.9)
NLR	
<2.2	340 (66.4)
≥2.2	172 (33.6)

SRC: signet-ring cell; SRCC: signet-ring cell carcinoma; pSRCC: pure signet-ring cell cancer; mSRCC: mixed signet-ring cell cancer; LM: lower and middle; MU: middle and upper; LMU: lower, middle, and upper; SII: systemic immune-inflammation index; PNI: prognostic nutritional index; NLR: lymphocyte to neutrophil ratio.

**Table 2:** The optimal cut-offs for prognostic factors according to ROC curves.

Variables	Threshold	Sensitivity	Specificity	AUC area (95% CI)	*p* value
Tumor size	4.75	0.744	0.385	0.735 (0.692-0.778)	<0.001
Leukocyte	6.17	0.459	0.404	0.502 (0.452-0.552)	0.941
Neutrophil	3.27	0.537	0.452	0.539 (0.489-0.589)	0.130
Hemoglobin	121.2	0.793	0.653	0.578 (0.528-0.627)	0.002
Fibrinogen	3.06	0.583	0.381	0.617 (0.569-0.665)	<0.001
Prealbumin	234.5	0.637	0.384	0.662 (0.616-0.709)	<0.001
Albumin	42.5	0.500	0.351	0.587 (0.538-0.637)	0.001
Globulin	29.9	0.194	0.148	0.505 (0.454-0.555)	0.855
SII	527	0.438	0.244	0.615 (0.566-0.664)	<0.001
NLR	2.2	0.426	0.256	0.593 (0.544-0.643)	<0.001
PNI	48.73	0.752	0.550	0.615 (0.566-0.663)	<0.001

ROC: receiver operating characteristic; AUC: area under curve; CI: confidence interval; SII: systemic immune-inflammation index; PNI: prognostic nutritional index; NLR: lymphocyte to neutrophil ratio.

**Table 3:** The correlation between the SII and other clinicopathological parameters.

Variables	SII < 527 (cases)	SII ≥ 527 (cases)	*p* value
Total			
Sex			0.062
Men	230	102	
Women	110	70	
Age (years)			0.414
<55	159	87	
≥55	181	85	
SRCC			0.384
mSRCC	298	146	
pSRCC	42	26	
Chemotherapy			0.352
Yes	167	77	
No	173	95	
Tumor size (cm)			<0.001
<4.75	175	53	
≥4.75	165	119	
pT			<0.001
T_1_	74	10	
T_2_	45	19	
T_3_	39	23	
T_4_	182	120	
pN			<0.001
N_0_	127	39	
N_1_	60	26	
N_2_	67	33	
N_3_a	60	42	
N_3_b	26	32	
pTNM			<0.001
I+II	173	47	
III	167	125	
Tumor location			0.079
Lower stomach	227	101	
Middle stomach	49	26	
Upper stomach	25	10	
LM stomach	29	24	
MU stomach	2	5	
LMU stomach	8	6	
Leukocyte			<0.001
<6.17	230	62	
≥6.17	110	110	
Neutrophil			<0.001
<3.27	231	29	
≥3.27	109	143	
Hemoglobin			<0.001
<121.2	60	80	
≥121.2	280	92	
Fibrinogen			<0.001
<3.06	204	64	
≥3.06	136	108	
Prealbumin			0.005
<234.5	149	98	
≥234.5	191	74	
Albumin			0.117
<42.5	187	82	
≥42.5	153	90	
Globulin			0.292
<29.9	278	147	
≥29.9	62	25	
PNI			<0.001
<48.73	92	84	
≥48.73	248	88	
Total gastrectomy			0.112
Yes	68	45	
No	272	127	
NLR			<0.001
<2.2	303	37	
≥2.2	37	135	

SII: systemic immune-inflammation index; SRCC: signet-ring cell carcinoma; pSRCC: pure signet-ring cell cancer; mSRCC: mixed signet-ring cell cancer; LM: lower and middle; MU: middle and upper; LMU: lower, middle, and upper; PNI: prognostic nutritional index; NLR: lymphocyte to neutrophil ratio.

**Table 4:** Analysis of prognostic factors in 512 patients with gastric cancer with an SRC component.

Variables	Survival analysis	
	5-YSR (%)	*p* value
Sex		
Male	53.3	0.639
Female	51.7	
Age (years)		
<55	58.5	0.007
≥55	47.4	
Tumor size (cm)		
<4.75	72.8	<0.001
≥4.75	36.6	
Chemotherapy		
Yes	56.6	0.059
No	49.3	
SRCC		
mSRCC	53.6	0.432
pSRCC	47.1	
T-stage		
T_1_	97.6	<0.001
T_2_	82.8	
T_3_	50.0	
T_4_	34.4	
N-stage		
N_0_	83.1	<0.001
N_1_	55.8	
N_2_	47.0	
N_3a_	30.4	
N_3b_	10.3	
TNM		
I+II	80.9	<0.001
III	31.5	
Tumor location		
L	59.5	<0.001
M	49.3	
U	37.1	
LM	37.7	
MU	28.6	
LMU	21.4	
Leukocyte		
<6.17	55.1	0.229
≥6.17	49.5	
Neutrophil		
<3.27	56.9	0.046
≥3.27	48.4	
Hemoglobin		
<121.2	40.0	<0.001
≥121.2	57.5	
Fibrinogen		
<3.06	62.3	<0.001
≥3.06	42.2	
Prealbumin		
<234.5	39.7	<0.001
≥234.5	64.9	
Albumin		
<42.5	46.2	<0.001
≥42.5	61.4	
Globulin		
<29.9	54.1	0.170
≥29.9	46.0	
NLR		
<2.2	59.1	0.006
≥2.2	40.1	
PNI		
<48.73	38.1	<0.001
≥48.73	60.4	
SII		
<527	60.0	<0.001
≥527	38.4	
Total gastrectomy		
Yes	22.1	<0.001
No	61.4	

SII: systemic immune-inflammation index; SRCC: signet-ring cell carcinoma; pSRCC: pure signet-ring cell cancer; mSRCC: mixed signet-ring cell cancer; LM: lower and middle; MU: middle and upper; LMU: lower, middle, and upper; PNI: prognostic nutritional index; NLR: lymphocyte to neutrophil ratio.

**Table 5:** Analysis of prognostic factors in 512 patients with gastric cancer with an SRC component by multivariate analysis.

	B	SE	Sig	Exp(B)	95% CI for Exp(B)	
					Lower	Upper
T_COV_	-0.003	0.006	0.652	0.997	0.986	1.009
T_COV_1	-0.008	0.006	0.167	0.992	0.981	1.003
T-stage	0.688	0.126	<0.001	1.989	1.554	2.546
N-stage	0.413	0.075	<0.001	1.512	1.305	1.751
TNM stage	-0.241	0.269	0.370	0.786	0.464	1.331
Tumor size	0.134	0.163	0.410	1.143	0.831	1.573
Age	0.200	0.139	0.151	1.221	0.930	1.603
Neutrophil	-0.221	0.176	0.209	0.802	0.568	1.132
NLR	0.000	0.186	0.999	1.000	0.695	1.438
Hemoglobin	-0.001	0.151	0.996	0.999	0.744	1.342
SII	0.523	0.197	0.008	1.686	1.146	2.481
PNI	0.020	0.192	0.916	1.020	0.701	1.486
Albumin	-0.128	0.188	0.498	0.880	0.609	1.273
Total gastrectomy	-0.809	0.180	<0.001	0.446	0.313	0.633
Tumor location LMU			0.436			
Tumor location L	0.431	0.352	0.222	1.538	0.771	3.068
Tumor location M	0.276	0.360	0.444	1.317	0.651	2.665
Tumor location U	0.650	0.384	0.091	1.915	0.901	4.068
Tumor location LM	0.136	0.361	0.707	1.145	0.565	2.323
Tumor location MU	0.384	0.561	0.494	1.468	0.489	4.405

SII: systemic immune-inflammation index; LM: lower and middle; MU: middle and upper; LMU: lower, middle, and upper; PNI: prognostic nutritional index; NLR: lymphocyte to neutrophil ratio; T_COV_: fibrinogen time-dependent variable; T_COV_1: prealbumin time-dependent variable.

## Data Availability

No data were used to support this study.
